# Bis(μ-dimesitylborinato-κ^2^ *O*:*O*)bis­[(2-methyl­pyridine-κ*N*)lithium]

**DOI:** 10.1107/S1600536808042748

**Published:** 2009-01-14

**Authors:** K. T. Pillai Saravana, Jung-Ho Son, James D. Hoefelmeyer

**Affiliations:** a Department of Chemistry, The University of South Dakota, 414 E. Clark St, Vermillion, SD 57069, USA

## Abstract

The title compound, [Li_2_(C_18_H_22_BO)_2_(C_6_H_7_N)_2_], is a lithium dimesitylboroxide dimer in which the lithium cation is also coordinated by one mol­ecule of 2-methyl­pyridine. At the core of the structure is an Li_2_O_2_ four-membered ring. The structure is centrosymmetric with an inversion centre midway between two Li atoms. Inter­molecular C—H⋯π inter­actions and π–π inter­actions between the 2-methyl­pyridine rings exist [centroid–centroid distance = 3.6312 (16) Å].

## Related literature

For related structures, see: Weese *et al.* (1987 ▶); Gibson *et al.* (1997 ▶); Cole *et al.* (2004 ▶).
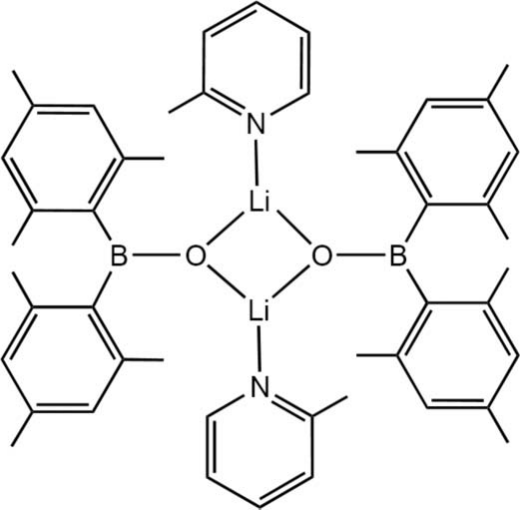


## Experimental

### 

#### Crystal data

[Li_2_(C_18_H_22_BO)_2_(C_6_H_7_N)_2_]
*M* *_r_* = 730.46
Monoclinic, 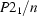

*a* = 8.6075 (11) Å
*b* = 9.1307 (11) Å
*c* = 26.220 (3) Å
β = 90.124 (2)°
*V* = 2060.7 (4) Å^3^
*Z* = 2
Mo *K*α radiation
μ = 0.07 mm^−1^
*T* = 100 (2) K
0.63 × 0.19 × 0.14 mm

#### Data collection

Bruker APEXII diffractometer
Absorption correction: multi-scan (*SADABS*; Bruker, 2006 ▶) *T*_min_ = 0.958, *T*_max_ = 0.990
8145 measured reflections
2848 independent reflections
2011 reflections with *I* > 2σ(*I*)
*R*_int_ = 0.036
θ_max_ = 23.4°

#### Refinement

*R*[*F* ^2^ > 2σ(*F* ^2^)] = 0.043
*wR*(*F* ^2^) = 0.140
*S* = 1.26
2848 reflections
260 parameters
H-atom parameters constrained
Δρ_max_ = 0.30 e Å^−3^
Δρ_min_ = −0.24 e Å^−3^

### 

Data collection: *APEX2* (Bruker, 2006 ▶); cell refinement: *SAINT* (Bruker, 2006 ▶); data reduction: *SAINT*; program(s) used to solve structure: *SHELXTL* (Sheldrick, 2008 ▶); program(s) used to refine structure: *SHELXTL*; molecular graphics: *ORTEP-3* (Farrugia, 1997 ▶) and *PLATON* (Spek, 2003 ▶); software used to prepare material for publication: *SHELXTL*.

## Supplementary Material

Crystal structure: contains datablocks I, global. DOI: 10.1107/S1600536808042748/gw2055sup1.cif

Structure factors: contains datablocks I. DOI: 10.1107/S1600536808042748/gw2055Isup2.hkl

Additional supplementary materials:  crystallographic information; 3D view; checkCIF report

## Figures and Tables

**Table 1:** Hydrogen-bond geometry (Å, °)

*D*—H⋯*A*	*D*—H	H⋯*A*	*D*⋯*A*	*D*—H⋯*A*
C5—H5⋯*Cg*1^i^	0.95	2.96	3.787 (3)	146

Symmetry code: (i) 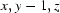. *Cg*1 is the centroid of the C8–C13 ring.

**Fig. 1.**
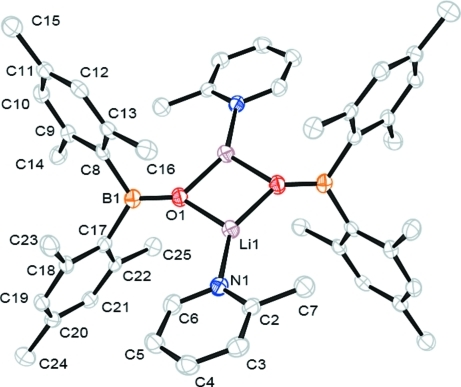
ORTEP drawing of Bis-µ2-[(2-methylpyridine)lithium(I)]-bis(dimesitylboroxide). Unlabled atoms are related with labeled part by inversion symmetry. Displacement ellipsoids are drawn at the 50% probability level. H atoms are omitted for clarity.

**Fig. 2.**
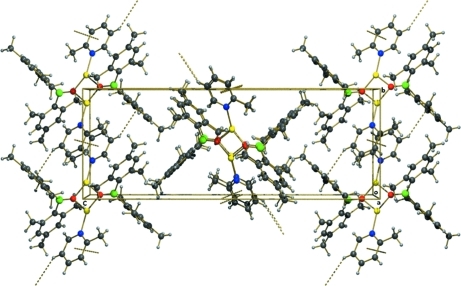
Packing diagram of Bis-µ2-[(2-methylpyridine)lithium(I)]-bis(dimesitylboroxide) viewed along a axis. Dotted lines show C— H···π and π–π interactions.

## supplementary crystallographic information

### Comment

The title compound is structurally similar to the compounds reported in the literature (Weese *et al.*, 1987; Gibson *et al.*, 1997; Cole *et al.*, 2004). The molecule contains a staggered conformation of C—B—C planes about the Li_2_O_2_ core, and pyramidalization about the three coordinate lithium(I).

The title compound consist of a lithium(I) cation coordinated to two dimesitylboroxide anions (Li1—O1 = 1.853 (5) Å and Li1—O1^i^ = 1.873 (5) Å) [symmetry code: (i) -*x* + 1, -*y* + 2, -*z*] and one molecule of 2-methylpyridine (Li1—N1 = 2.084 (5) Å). The environment around boron is distorted trigonal planar (sum of the angles around B1 = 360.0°). The asymmetric units are joined *via* planar Li_2_O_2_ four member ring (Li1···Li1^i^ = 2.444 (8) Å). The lithium cation is three-coordinate and slightly pyramidalized (sum of the angles around Li1 = 356.03°). The O1—Li1—O1^i^ and O1^i^—Li1—N1 angles deviate from an ideal trigonal planar geometry (98.0 (2) ° and 138.6 (3) °, respectively), with the expanded angle a result of steric repulsion between the methyl group (C7) of 2-methylpyridine and dimesitylboroxide group of the adjoining asymmetric unit. The mean plane of boron triangle forms a 48.96 (16) ° angle against the Li_2_O_2_ plane. The crystal structure contains intermolecular C5—H5···*Cg*1^ii^ interaction with H···*Cg* = 2.96 Å, C—H···*Cg* angle 146° and C···Cg = 3.787 (3) Å (*Cg*1 is centroid of C8—C13) [symmetry code: (ii) *x*, -1 + *y*, *z*]. Intermolecular face to face π–π interaction between the 2-methylpyridine rings occurs with *Cg*2···*Cg*2^iii^ = 3.6312 (`6) Å (*Cg*2 is centroid of N1—C6) [symmetry code: (iii) -*x* + 1, -*y* + 1, -*z*].

### Experimental

To a solution of 2-methylpyridine (0.45 ml, 3.8 mmol), n-butyllithium (1.6 *M*, 2.5 ml, 4.2 mmol) in 10 ml hexane was added dropwise through an addition funnel at -78 °C under inert atmosphere. The resulting red color solution was stirred for 30 minutes at -78 °C. Meanwhile, dimesitylboronfluoride (1.0 g, 3.8 mmol) was dissolved in hexane (10 ml) in a round bottom flask and kept under nitrogen atmosphere. The dimesitylboronfluoride solution was transferred to the organolithium by cannula. The resultant yellow color solution was stirred (18 h) under nitrogen atmosphere. The product was filtered through a frit and a yellowish precipitate formed upon exposure to air. The product was dissolved in toluene, and crystals were recovered upon slow evaporation of the solvent.

### Refinement

C-bound H atoms were positioned geometrically with C—H (aromatic) = 0.95 Å and C—H (methyl) = 0.98 Å and allowed to ride on the parent atoms with *U*_iso_(H) = 1.2Ueq(C) and *U*_iso_(H) = 1.5Ueq(C), respectively.

### Crystal data

**Table:**

[Li_2_(C_18_H_22_BO)_2_(C_6_H_7_N)_2_]	*F*(000) = 784
*M**_r_* = 730.46	*D*_x_ = 1.177 Mg m^−^^3^
Monoclinic, *P*2_1_/*n*	Mo *K*α radiation, λ = 0.71073 Å
Hall symbol: -P 2yn	Cell parameters from 1946 reflections
*a* = 8.6075 (11) Å	θ = 2.5–23.1°
*b* = 9.1307 (11) Å	µ = 0.07 mm^−^^1^
*c* = 26.220 (3) Å	*T* = 100 K
β = 90.124 (2)°	Needle, colorless
*V* = 2060.7 (4) Å^3^	0.63 × 0.19 × 0.14 mm
*Z* = 2	

### Data collection

**Table:**

Bruker SMART APEXII diffractometer	2848 independent reflections
Radiation source: fine-focus sealed tube	2011 reflections with *I* > 2σ(*I*)
graphite	*R*_int_ = 0.036
ω scans	θ_max_ = 23.4°, θ_min_ = 1.6°
Absorption correction: multi-scan (*SADABS*; Bruker, 2006)	*h* = −9→9
*T*_min_ = 0.958, *T*_max_ = 0.990	*k* = −10→9
8145 measured reflections	*l* = −28→29

### Refinement

**Table:**

Refinement on *F*^2^	Primary atom site location: structure-invariant direct methods
Least-squares matrix: full	Secondary atom site location: difference Fourier map
*R*[*F*^2^ > 2σ(*F*^2^)] = 0.043	Hydrogen site location: inferred from neighbouring sites
*wR*(*F*^2^) = 0.140	H-atom parameters constrained
*S* = 1.26	*w* = 1/[σ^2^(*F*_o_^2^) + (0.1*P*)^2^] where *P* = (*F*_o_^2^ + 2*F*_c_^2^)/3
2848 reflections	(Δ/σ)_max_ = 0.001
260 parameters	Δρ_max_ = 0.30 e Å^−^^3^
0 restraints	Δρ_min_ = −0.24 e Å^−^^3^

### Special details

**Table:**

Geometry. All e.s.d.'s (except the e.s.d. in the dihedral angle between two l.s. planes) are estimated using the full covariance matrix. The cell e.s.d.'s are taken into account individually in the estimation of e.s.d.'s in distances, angles and torsion angles; correlations between e.s.d.'s in cell parameters are only used when they are defined by crystal symmetry. An approximate (isotropic) treatment of cell e.s.d.'s is used for estimating e.s.d.'s involving l.s. planes.
Refinement. Refinement of *F*^2^ against ALL reflections. The weighted *R*-factor *wR* and goodness of fit *S* are based on *F*^2^, conventional *R*-factors *R* are based on *F*, with *F* set to zero for negative *F*^2^. The threshold expression of *F*^2^ > σ(*F*^2^) is used only for calculating *R*-factors(gt) *etc*. and is not relevant to the choice of reflections for refinement. *R*-factors based on *F*^2^ are statistically about twice as large as those based on *F*, and *R*- factors based on ALL data will be even larger.

### Fractional atomic coordinates and isotropic or equivalent isotropic displacement parameters (Å^2^)

**Table:**

	*x*	*y*	*z*	*U*_iso_*/*U*_eq_	
C2	0.7338 (3)	0.5897 (3)	−0.00706 (10)	0.0223 (6)	
C3	0.7904 (3)	0.4572 (3)	0.01163 (10)	0.0263 (7)	
H3	0.8696	0.4059	−0.0062	0.032*	
C4	0.7299 (3)	0.4011 (3)	0.05639 (10)	0.0282 (7)	
H4	0.7681	0.3114	0.0699	0.034*	
C5	0.6140 (3)	0.4763 (3)	0.08128 (10)	0.0281 (7)	
H5	0.5699	0.4395	0.1119	0.034*	
C6	0.5637 (3)	0.6068 (3)	0.06045 (10)	0.0253 (7)	
H6	0.4831	0.6584	0.0774	0.030*	
C7	0.7948 (3)	0.6533 (3)	−0.05569 (10)	0.0297 (7)	
H7A	0.8423	0.5755	−0.0762	0.045*	
H7B	0.8730	0.7281	−0.0478	0.045*	
H7C	0.7092	0.6979	−0.0749	0.045*	
C8	0.4570 (3)	1.0810 (3)	0.13862 (9)	0.0197 (6)	
C9	0.3566 (3)	1.1589 (3)	0.17090 (9)	0.0208 (6)	
C10	0.4168 (3)	1.2560 (3)	0.20712 (9)	0.0236 (6)	
H10	0.3469	1.3111	0.2275	0.028*	
C11	0.5757 (3)	1.2744 (3)	0.21418 (10)	0.0218 (6)	
C12	0.6750 (3)	1.1964 (3)	0.18272 (10)	0.0232 (7)	
H12	0.7841	1.2070	0.1869	0.028*	
C13	0.6178 (3)	1.1022 (3)	0.14482 (10)	0.0222 (6)	
C14	0.1828 (3)	1.1379 (3)	0.16953 (10)	0.0276 (7)	
H14A	0.1558	1.0466	0.1870	0.041*	
H14B	0.1475	1.1331	0.1340	0.041*	
H14C	0.1322	1.2204	0.1867	0.041*	
C15	0.6359 (3)	1.3743 (3)	0.25549 (10)	0.0304 (7)	
H15A	0.7469	1.3562	0.2608	0.046*	
H15B	0.5796	1.3552	0.2872	0.046*	
H15C	0.6202	1.4765	0.2453	0.046*	
C16	0.7350 (3)	1.0196 (3)	0.11262 (10)	0.0272 (7)	
H16A	0.7170	0.9141	0.1160	0.041*	
H16B	0.8403	1.0431	0.1243	0.041*	
H16C	0.7233	1.0483	0.0768	0.041*	
C17	0.2773 (3)	0.8374 (3)	0.11617 (9)	0.0214 (6)	
C18	0.3143 (3)	0.7485 (3)	0.15886 (9)	0.0227 (6)	
C19	0.2096 (3)	0.6436 (3)	0.17580 (10)	0.0230 (7)	
H19	0.2383	0.5830	0.2037	0.028*	
C20	0.0641 (3)	0.6238 (3)	0.15342 (10)	0.0235 (7)	
C21	0.0300 (3)	0.7095 (3)	0.11096 (10)	0.0228 (6)	
H21	−0.0675	0.6971	0.0945	0.027*	
C22	0.1331 (3)	0.8122 (3)	0.09178 (9)	0.0212 (6)	
C23	0.4655 (3)	0.7641 (3)	0.18735 (10)	0.0312 (7)	
H23A	0.4646	0.8554	0.2070	0.047*	
H23B	0.5517	0.7662	0.1630	0.047*	
H23C	0.4787	0.6809	0.2106	0.047*	
C24	−0.0507 (3)	0.5159 (3)	0.17434 (11)	0.0358 (8)	
H24A	−0.0458	0.4250	0.1545	0.054*	
H24B	−0.1556	0.5571	0.1721	0.054*	
H24C	−0.0256	0.4951	0.2101	0.054*	
C25	0.0840 (3)	0.8989 (3)	0.04561 (10)	0.0276 (7)	
H25A	−0.0198	0.8675	0.0348	0.041*	
H25B	0.1581	0.8824	0.0179	0.041*	
H25C	0.0817	1.0034	0.0542	0.041*	
Li1	0.5311 (5)	0.8695 (5)	−0.00153 (16)	0.0253 (10)	
B1	0.3913 (3)	0.9657 (3)	0.09683 (11)	0.0203 (7)	
N1	0.6228 (3)	0.6653 (2)	0.01732 (8)	0.0236 (6)	
O1	0.43136 (19)	0.97903 (18)	0.04806 (6)	0.0246 (5)	

### Atomic displacement parameters (Å^2^)

**Table:**

	*U*^11^	*U*^22^	*U*^33^	*U*^12^	*U*^13^	*U*^23^
C2	0.0251 (16)	0.0196 (15)	0.0222 (15)	0.0003 (12)	0.0001 (13)	−0.0035 (12)
C3	0.0253 (16)	0.0231 (15)	0.0306 (16)	0.0050 (13)	0.0003 (13)	−0.0029 (13)
C4	0.0328 (17)	0.0207 (15)	0.0312 (17)	0.0029 (13)	−0.0033 (14)	0.0031 (13)
C5	0.0326 (17)	0.0260 (16)	0.0258 (16)	−0.0022 (13)	−0.0004 (14)	0.0043 (13)
C6	0.0231 (16)	0.0281 (16)	0.0246 (16)	0.0015 (13)	0.0027 (13)	−0.0027 (13)
C7	0.0296 (16)	0.0274 (16)	0.0320 (17)	0.0042 (13)	0.0060 (13)	0.0009 (13)
C8	0.0238 (15)	0.0167 (13)	0.0186 (14)	0.0002 (12)	0.0031 (12)	0.0072 (11)
C9	0.0209 (15)	0.0236 (15)	0.0181 (14)	0.0007 (12)	0.0018 (12)	0.0030 (12)
C10	0.0266 (16)	0.0229 (14)	0.0214 (15)	0.0038 (13)	0.0047 (12)	0.0024 (12)
C11	0.0233 (16)	0.0215 (15)	0.0205 (14)	−0.0017 (12)	0.0001 (13)	0.0046 (12)
C12	0.0225 (15)	0.0219 (14)	0.0253 (15)	−0.0031 (12)	0.0009 (13)	0.0088 (12)
C13	0.0244 (16)	0.0186 (14)	0.0235 (15)	0.0014 (12)	0.0053 (12)	0.0068 (12)
C14	0.0252 (16)	0.0308 (16)	0.0267 (16)	0.0022 (13)	0.0037 (13)	−0.0041 (13)
C15	0.0308 (17)	0.0310 (16)	0.0293 (16)	−0.0039 (13)	0.0005 (14)	0.0018 (13)
C16	0.0207 (15)	0.0257 (15)	0.0352 (16)	0.0009 (12)	0.0047 (13)	0.0008 (13)
C17	0.0276 (16)	0.0188 (14)	0.0176 (14)	0.0042 (12)	0.0042 (12)	−0.0023 (11)
C18	0.0299 (17)	0.0207 (14)	0.0175 (14)	0.0030 (13)	0.0042 (12)	0.0014 (12)
C19	0.0289 (16)	0.0201 (14)	0.0200 (14)	0.0028 (13)	0.0040 (13)	0.0026 (11)
C20	0.0256 (16)	0.0204 (15)	0.0245 (15)	−0.0010 (12)	0.0083 (13)	−0.0031 (12)
C21	0.0199 (15)	0.0243 (15)	0.0243 (15)	0.0031 (12)	0.0026 (12)	−0.0041 (12)
C22	0.0275 (16)	0.0195 (14)	0.0167 (14)	0.0034 (12)	0.0045 (12)	−0.0047 (11)
C23	0.0387 (18)	0.0306 (16)	0.0242 (15)	−0.0017 (14)	−0.0066 (14)	0.0074 (13)
C24	0.0365 (18)	0.0321 (17)	0.0387 (17)	−0.0080 (14)	0.0045 (15)	0.0021 (14)
C25	0.0262 (16)	0.0302 (16)	0.0264 (15)	0.0024 (13)	−0.0014 (13)	−0.0006 (13)
Li1	0.026 (3)	0.025 (2)	0.025 (2)	−0.003 (2)	0.005 (2)	−0.0003 (19)
B1	0.0181 (17)	0.0217 (16)	0.0211 (17)	0.0092 (13)	0.0021 (14)	0.0010 (13)
N1	0.0270 (13)	0.0230 (12)	0.0208 (13)	0.0008 (10)	0.0007 (11)	−0.0012 (10)
O1	0.0290 (11)	0.0235 (10)	0.0212 (11)	0.0030 (8)	0.0036 (9)	0.0023 (8)

### Geometric parameters (Å, °)

**Table:**

C2—N1	1.342 (3)	C15—H15C	0.9800
C2—C3	1.393 (4)	C16—H16A	0.9800
C2—C7	1.497 (4)	C16—H16B	0.9800
C3—C4	1.383 (4)	C16—H16C	0.9800
C3—H3	0.9500	C17—C22	1.414 (4)
C4—C5	1.377 (4)	C17—C18	1.419 (3)
C4—H4	0.9500	C17—B1	1.611 (4)
C5—C6	1.380 (4)	C18—C19	1.389 (3)
C5—H5	0.9500	C18—C23	1.506 (4)
C6—N1	1.351 (3)	C19—C20	1.393 (4)
C6—H6	0.9500	C19—H19	0.9500
C7—H7A	0.9800	C20—C21	1.392 (4)
C7—H7B	0.9800	C20—C24	1.500 (4)
C7—H7C	0.9800	C21—C22	1.386 (4)
C8—C9	1.405 (3)	C21—H21	0.9500
C8—C13	1.406 (3)	C22—C25	1.506 (3)
C8—B1	1.620 (4)	C23—H23A	0.9800
C9—C10	1.398 (3)	C23—H23B	0.9800
C9—C14	1.509 (4)	C23—H23C	0.9800
C10—C11	1.390 (4)	C24—H24A	0.9800
C10—H10	0.9500	C24—H24B	0.9800
C11—C12	1.386 (4)	C24—H24C	0.9800
C11—C15	1.507 (4)	C25—H25A	0.9800
C12—C13	1.403 (4)	C25—H25B	0.9800
C12—H12	0.9500	C25—H25C	0.9800
C13—C16	1.517 (4)	Li1—O1	1.853 (5)
C14—H14A	0.9800	Li1—O1^i^	1.873 (5)
C14—H14B	0.9800	Li1—N1	2.084 (5)
C14—H14C	0.9800	Li1—Li1^i^	2.444 (8)
C15—H15A	0.9800	B1—O1	1.331 (3)
C15—H15B	0.9800	O1—Li1^i^	1.873 (5)
			
N1—C2—C3	121.8 (2)	C13—C16—H16C	109.5
N1—C2—C7	117.2 (2)	H16A—C16—H16C	109.5
C3—C2—C7	120.9 (3)	H16B—C16—H16C	109.5
C4—C3—C2	119.3 (3)	C22—C17—C18	117.3 (2)
C4—C3—H3	120.4	C22—C17—B1	120.7 (2)
C2—C3—H3	120.4	C18—C17—B1	121.9 (2)
C5—C4—C3	119.5 (2)	C19—C18—C17	120.2 (2)
C5—C4—H4	120.3	C19—C18—C23	117.9 (2)
C3—C4—H4	120.3	C17—C18—C23	122.0 (2)
C4—C5—C6	118.0 (3)	C18—C19—C20	122.6 (2)
C4—C5—H5	121.0	C18—C19—H19	118.7
C6—C5—H5	121.0	C20—C19—H19	118.7
N1—C6—C5	123.7 (3)	C21—C20—C19	116.8 (2)
N1—C6—H6	118.2	C21—C20—C24	121.6 (2)
C5—C6—H6	118.2	C19—C20—C24	121.6 (2)
C2—C7—H7A	109.5	C22—C21—C20	122.5 (2)
C2—C7—H7B	109.5	C22—C21—H21	118.8
H7A—C7—H7B	109.5	C20—C21—H21	118.8
C2—C7—H7C	109.5	C21—C22—C17	120.5 (2)
H7A—C7—H7C	109.5	C21—C22—C25	118.0 (2)
H7B—C7—H7C	109.5	C17—C22—C25	121.5 (2)
C9—C8—C13	117.9 (2)	C18—C23—H23A	109.5
C9—C8—B1	121.5 (2)	C18—C23—H23B	109.5
C13—C8—B1	120.6 (2)	H23A—C23—H23B	109.5
C10—C9—C8	120.2 (2)	C18—C23—H23C	109.5
C10—C9—C14	117.6 (2)	H23A—C23—H23C	109.5
C8—C9—C14	122.2 (2)	H23B—C23—H23C	109.5
C11—C10—C9	122.0 (3)	C20—C24—H24A	109.5
C11—C10—H10	119.0	C20—C24—H24B	109.5
C9—C10—H10	119.0	H24A—C24—H24B	109.5
C12—C11—C10	117.8 (2)	C20—C24—H24C	109.5
C12—C11—C15	121.8 (2)	H24A—C24—H24C	109.5
C10—C11—C15	120.3 (2)	H24B—C24—H24C	109.5
C11—C12—C13	121.4 (2)	C22—C25—H25A	109.5
C11—C12—H12	119.3	C22—C25—H25B	109.5
C13—C12—H12	119.3	H25A—C25—H25B	109.5
C12—C13—C8	120.7 (2)	C22—C25—H25C	109.5
C12—C13—C16	117.8 (2)	H25A—C25—H25C	109.5
C8—C13—C16	121.5 (2)	H25B—C25—H25C	109.5
C9—C14—H14A	109.5	O1—Li1—O1^i^	98.0 (2)
C9—C14—H14B	109.5	O1—Li1—N1	119.5 (2)
H14A—C14—H14B	109.5	O1^i^—Li1—N1	138.6 (3)
C9—C14—H14C	109.5	O1—Li1—Li1^i^	49.36 (16)
H14A—C14—H14C	109.5	O1^i^—Li1—Li1^i^	48.66 (16)
H14B—C14—H14C	109.5	N1—Li1—Li1^i^	161.4 (3)
C11—C15—H15A	109.5	O1—B1—C17	121.9 (2)
C11—C15—H15B	109.5	O1—B1—C8	120.0 (2)
H15A—C15—H15B	109.5	C17—B1—C8	118.1 (2)
C11—C15—H15C	109.5	C2—N1—C6	117.7 (2)
H15A—C15—H15C	109.5	C2—N1—Li1	128.1 (2)
H15B—C15—H15C	109.5	C6—N1—Li1	114.2 (2)
C13—C16—H16A	109.5	B1—O1—Li1	138.3 (2)
C13—C16—H16B	109.5	B1—O1—Li1^i^	137.6 (2)
H16A—C16—H16B	109.5	Li1—O1—Li1^i^	82.0 (2)
			
N1—C2—C3—C4	0.4 (4)	C18—C17—C22—C21	3.3 (4)
C7—C2—C3—C4	−179.3 (2)	B1—C17—C22—C21	−174.9 (2)
C2—C3—C4—C5	0.7 (4)	C18—C17—C22—C25	−179.0 (2)
C3—C4—C5—C6	−0.7 (4)	B1—C17—C22—C25	2.8 (4)
C4—C5—C6—N1	−0.5 (4)	C22—C17—B1—O1	−50.9 (3)
C13—C8—C9—C10	1.2 (3)	C18—C17—B1—O1	131.0 (3)
B1—C8—C9—C10	179.0 (2)	C22—C17—B1—C8	128.9 (3)
C13—C8—C9—C14	−176.5 (2)	C18—C17—B1—C8	−49.2 (3)
B1—C8—C9—C14	1.4 (3)	C9—C8—B1—O1	125.9 (3)
C8—C9—C10—C11	−2.9 (4)	C13—C8—B1—O1	−56.3 (3)
C14—C9—C10—C11	174.9 (2)	C9—C8—B1—C17	−53.9 (3)
C9—C10—C11—C12	2.2 (4)	C13—C8—B1—C17	123.9 (3)
C9—C10—C11—C15	−176.8 (2)	C3—C2—N1—C6	−1.6 (3)
C10—C11—C12—C13	0.2 (4)	C7—C2—N1—C6	178.1 (2)
C15—C11—C12—C13	179.1 (2)	C3—C2—N1—Li1	176.2 (2)
C11—C12—C13—C8	−1.8 (4)	C7—C2—N1—Li1	−4.1 (3)
C11—C12—C13—C16	−179.2 (2)	C5—C6—N1—C2	1.7 (4)
C9—C8—C13—C12	1.1 (3)	C5—C6—N1—Li1	−176.4 (2)
B1—C8—C13—C12	−176.8 (2)	O1—Li1—N1—C2	−159.0 (2)
C9—C8—C13—C16	178.4 (2)	O1^i^—Li1—N1—C2	−7.0 (5)
B1—C8—C13—C16	0.5 (3)	Li1^i^—Li1—N1—C2	−110.7 (10)
C22—C17—C18—C19	−1.2 (4)	O1—Li1—N1—C6	18.8 (3)
B1—C17—C18—C19	177.0 (2)	O1^i^—Li1—N1—C6	170.8 (3)
C22—C17—C18—C23	179.1 (2)	Li1^i^—Li1—N1—C6	67.1 (11)
B1—C17—C18—C23	−2.7 (4)	C17—B1—O1—Li1	−60.0 (4)
C17—C18—C19—C20	−2.1 (4)	C8—B1—O1—Li1	120.2 (3)
C23—C18—C19—C20	177.6 (2)	C17—B1—O1—Li1^i^	143.1 (3)
C18—C19—C20—C21	3.3 (4)	C8—B1—O1—Li1^i^	−36.6 (4)
C18—C19—C20—C24	−176.3 (2)	O1^i^—Li1—O1—B1	−164.5 (3)
C19—C20—C21—C22	−1.1 (4)	N1—Li1—O1—B1	−2.8 (5)
C24—C20—C21—C22	178.4 (2)	Li1^i^—Li1—O1—B1	−164.5 (3)
C20—C21—C22—C17	−2.1 (4)	O1^i^—Li1—O1—Li1^i^	0.0
C20—C21—C22—C25	−180.0 (2)	N1—Li1—O1—Li1^i^	161.7 (4)

Symmetry codes: (i) −*x*+1, −*y*+2, −*z*.

### Hydrogen-bond geometry (Å, °)

**Table:**

*D*—H···*A*	*D*—H	H···*A*	*D*···*A*	*D*—H···*A*
C5—H5···Cg1^ii^	0.95	2.96	3.787 (3)	146

Symmetry codes: (ii) *x*, *y*−1, *z*.
